# Enhancing stability and efficiency of perovskite solar cells with crosslinkable silane-functionalized and doped fullerene

**DOI:** 10.1038/ncomms12806

**Published:** 2016-10-05

**Authors:** Yang Bai, Qingfeng Dong, Yuchuan Shao, Yehao Deng, Qi Wang, Liang Shen, Dong Wang, Wei Wei, Jinsong Huang

**Affiliations:** 1 Department of Mechanical and Materials Engineering, College of Engineering, University of Nebraska-Lincoln, Lincoln, Nebraska 68588-0656, USA; 2 Nebraska Center for Materials and Nanoscience, University of Nebraska-Lincoln, Lincoln, Nebraska 68588-0656, USA

## Abstract

The instability of hybrid perovskite materials due to water and moisture arises as one major challenge to be addressed before any practical application of the demonstrated high efficiency perovskite solar cells. Here we report a facile strategy that can simultaneously enhance the stability and efficiency of p–i–n planar heterojunction-structure perovskite devices. Crosslinkable silane molecules with hydrophobic functional groups are bonded onto fullerene to make the fullerene layer highly water-resistant. Methylammonium iodide is introduced in the fullerene layer for n-doping via anion-induced electron transfer, resulting in dramatically increased conductivity over 100-fold. With crosslinkable silane-functionalized and doped fullerene electron transport layer, the perovskite devices deliver an efficiency of 19.5% with a high fill factor of 80.6%. A crosslinked silane-modified fullerene layer also enhances the water and moisture stability of the non-sealed perovskite devices by retaining nearly 90% of their original efficiencies after 30 days' exposure in an ambient environment.

## Abstract (web summary)

Perovskite solar cells reach high efficiencies but their stability remains a challenge. Here, Bai *et al*. functionalize the fullerene-based transport layer with hydrophobic and crosslinkable molecules to prepare devices reaching 19% efficiency and degrading by 10% over a month in ambient conditions.

## Graphical abstract

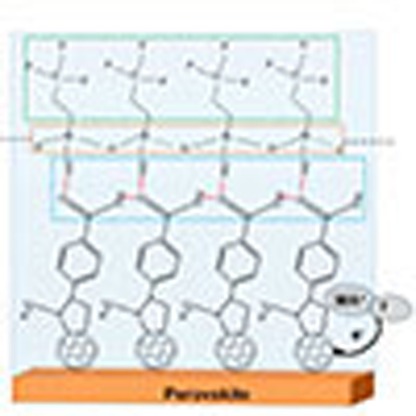

Organic–inorganic halide perovskite materials as light harvesters for new-generation photovoltaic application have been attracting tremendous attention in both scientific and industrial communities in the past few years^1^,^2^,^3^,^4^,^5^,^6^,^7^,^8^,^9^. The certified power conversion efficiency has skyrocketed from 3.8 to 22.1% (http://www.nrel.gov/ncpv/images/efficiency_chart.jpg.), owing to the material's intriguing optoelectronic properties such as its high absorption coefficient^10^, its high charge carrier mobility and lifetime^11^,^12^, and its long carrier diffusion length^13^,^14^,^15^. A variety of perovskite photovoltaic device architectures have been designed ranging from mesoscopic to planar structures with n–i–p or p–i–n layouts^6^,^10^,^16^,^17^,^18^. Although the highest efficiency obtained in mesoscopic type devices is already higher than the commercial CIGS and CdTe thin film solar cells, the intrinsic instability of perovskite devices owing to moisture and water hampers their practical application in ambient conditions^19^,^20^,^21^. Recently, a number of endeavours have been directed at improving the long-term stability of perovskite devices. One strategy is to develop new two-dimensional layered perovskite materials, such as (C_6_H_5_(CH_2_)_2_NH_3_)_2_(CH_3_NH_3_)_2_[Pb_3_I_10_]. Though this layered perovskite film showed enhanced moisture resistivity, the enlarged bandgap and exciton binding energy resulted in a low power conversion efficiency (*PCE*) of only 4.4% (ref. 22). Applying a water-resisting layer on the surface of perovskite film was demonstrated as another effective approach to enhance the moisture resistance of regular perovskite devices. Alkylphosphonic acid ω-ammonium, polyethylene glycol, tertiary and quaternary hydrophobic alkyl ammonium cations have been assembled in the bulk or on the surface of perovskite film as a moisture-resisting layer, yielding device efficiency in the range of 15.0–16.0% (refs 23, 24, 25). Though the moisture stability of perovskite film indeed was improved in those designs, the widely used spiro-OMeTAD hole transport layers (HTLs) still limit the device stability due to the moisture affinity of Li salt dopants^19^,^20^,^21^. Thus there is growing interest in a p–i–n planar heterojunction structure, which generally contains fullerene (typically (6,6]-phenyl-C_61_-butyric acid methyl ester (PCBM)) as the electron transport/collection layers (ETLs) because of several advantages they have such as photocurrent-hysteresis-free devices, low-temperature processing and high device efficiency^17^,^18^,^26^,^27^. Unfortunately, the relatively thin fullerene layer compared with the thick spiro-OMeTAD layer is not sufficient to effectively protect the perovskite layer from moisture damage in a humid environment. To tackle this issue, You *et al*.^28^ replaced fullerene with thicker ZnO ETL in p–i–n perovskite devices. Enhanced device stability was demonstrated, but the average device performance of *∼*15.0% is still far inferior to their fullerene counterpart^17^,^27^. More recently, an inorganic Ti(Nb)O_*x*_ buffer layer has been introduced on top of the PCBM layer to improve the device stability^29^. The judicious control of the heavily doped inorganic layer may induce much complexity for practical production. Therefore, it is still essential and urgent to develop a facile route for enhancing the moisture resistance of perovskite devices without sacrificing photovoltaic performance.

In this manuscript, we report a water-resistant crosslinkable silane-functionalized fullerene ETL to improve the moisture stability of p–i–n planar perovskite solar cells. We also introduce doping to the crosslinked silane-modified fullerene layer so that its conductivity is not compromised by the crosslinking process. The combination of crosslinking and doping has resulted in both high efficiency and stable perovskite solar cells in an ambient environment without resorting to encapsulation techniques.

## Results

### Formation of water-resistant fullerene layer

**Figure 1:**
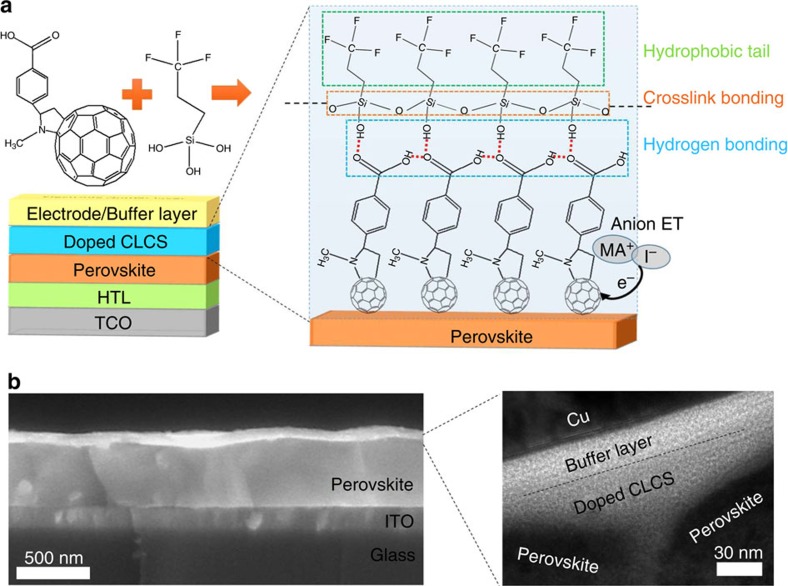
Scheme and structure of perovskite solar cell. (**a**) Device structure of the perovskite planar heterojunction solar cells and schematic illustration for the crosslinking of C_60_-SAM with silane-coupling agent. ET, electron transfer. (**b**) Cross-section SEM image of a typical perovskite device with doped crosslinked C_60_-SAM (CLCS) ETL and high-resolution transmission electron microscopy (TEM) image of the cross-section area of the doped CLCS ETL.

The concept of crosslinking fullerene is illustrated in Fig. 1a. The planar heterojunction p–i–n perovskite solar cells studied in this work have a structure of transparent conductive electrodes (TCE)/hole transport layer (HTL)/perovskite/ ETL/top electrode. Here C_60_-substituted benzoic acid self-assembled monolayer (C_60_-SAM) whose chemical structure is shown in the Fig. 1a was applied to form hydrogen bonding with the crosslinking agent. Trichloro(3,3,3-trifluoropropyl)silane, which readily hydrolyses to form three hydroxyl groups (–OH), was selected as the crosslinking agent. The carboxyl group (–COOH) of the C_60_-SAM material easily formed a strong hydrogen bond with one of the hydroxyl groups on silane, and the silicon-oxygen (Si–O) bonds generated from the silane-coupling reaction crosslinked the C_60_-SAM and silane molecules together. In addition, the trifluoromethyl groups (–CF_3_) from the silane materials made the crosslinked C_60_-SAM layer more hydrophobic, which prevented the moisture and water from penetrating into the perovskite layer. Therefore, the crosslinked C_60_-SAM layer acted not only as the electron transport and passivation layer, but also as a water-resistant layer to protect the perovskite film against damage by moisture.

To fabricate the perovskite devices, the CH_3_NH_3_PbI_3_ (MAPbI_3_) perovskite films were prepared by the two-step interdiffusion method^30^, where lead iodide (PbI_2_) and methylammonium iodide (CH_3_NH_3_I, MAI) were sequentially spun onto the poly(bis(4-phenyl)(2,4,6-trimethylphenyl)amine) (PTAA)-covered indium tin oxide (ITO) substrates, followed by a solvent annealing process^10^. A smooth and compact perovskite film was obtained with an average grain size much larger than the thickness of the films^27^. C_60_-SAM was then spun on top of the perovskite films and thermally annealed at 100 °C for 60 min. To crosslink C_60_-SAM, 5–8 μl of trichloro(3,3,3-trifluoropropyl)silane was added at the edge of the 5 inch diameter petri dishes during the thermal annealing process. The silane vapour was expected to penetrate into and crosslink the C_60_-SAM layer. The devices were completed by thermal evaporation of a buffer layer and a top electrode. More detailed information on film and device fabrication can be found in the experiment section. Figure 1b shows a typical cross-section scanning electron microscopy (SEM) image of a device. To find out the accurate thickness of the crosslinked C_60_-SAM layer, high-resolution transmission electron microscopy of the cross-sectional area of the film was carried out. The thickness of the optimized crosslinked C_60_-SAM layer was found to be roughly 20 nm.

**Figure 2:**
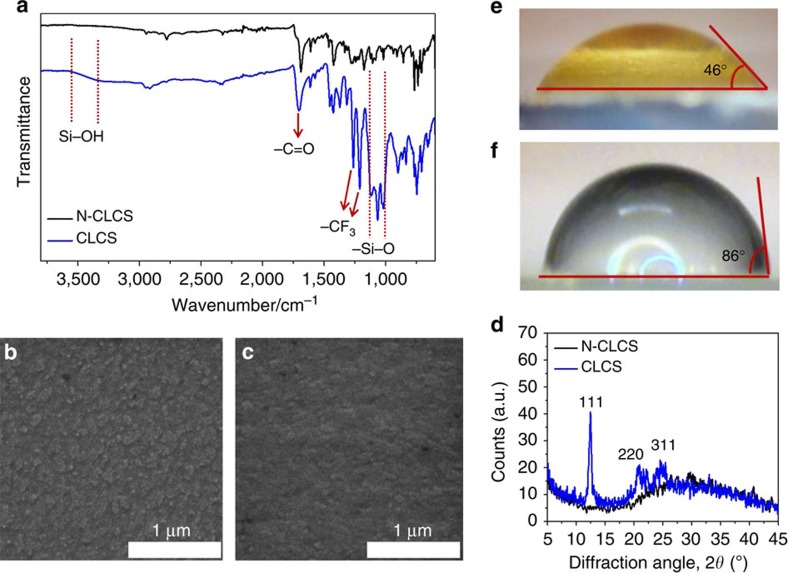
Characterization of N-CLCS and CLCS films. (**a**) FTIR spectra, (**b**,**c**) SEM images and (**d**) X-ray diffraction patterns of the N-CLCS and CLCS films. (**e**,**f**) The contact angles of water on N-CLCS/perovskite film and on CLCS/perovskite film. N-CLCS, non-crosslinked C_60_-SAM; CLCS, crosslinked C_60_-SAM.

Fourier transform infrared spectroscopy (FTIR) transmission spectra of C_60_-SAM before and after silane modification were collected to verify the formation of crosslinked C_60_-SAM. C_60_-SAM films on ITO substrates with and without silane modification were prepared by following the same procedure as that for device fabrication but with larger thickness, and the collected powder was subjected to FTIR analysis. As shown in Fig. 2a, several new bands clearly appeared in the corresponding FTIR spectrum after crosslinking with the silane agent. The strong vibration bands at 1,262 and 1,209 cm^−1^ can be ascribed to the –CF_3_ groups added to C_60_-SAM. As illustrated in Fig. 1, we speculate that one free –OH group of silane molecule forms a hydrogen bond with the –COOH group of C_60_-SAM, and the other two –OH groups are involved in the silane coupling reaction to form a Si–O–Si bond. The strong and wide vibration peaks (1,126–1,002 cm^−1^) shown in Fig. 2a confirm the formation of Si–O–Si bonds^31^,^32^. Though the hydrogen bonds of Si–OH···–COOH do not directly correspond to a vibration peak in the FTIR spectrum, they should affect the Si–OH and C=O chemical bonds by increasing their vibration frequency. Previous studies^33^ indicate that the isolated Si–OH groups show a sharp band at 3,690 cm^−1^. When the silane agent crosslinked with the C_60_-SAM layer via hydrogen bonding, the C=O vibration band moved from 1,686 cm^−1^ to a higher wavenumber of 1,705 cm^−1^. In addition, the appearance of a broad band at frequency region of 3,400–3,200 cm^−1^ indicates that the Si–OH groups are hydrogen-bonded^33^. These results demonstrate well that the C_60_-SAM layer was successfully crosslinked with the silane coupling agent through hydrogen bonding.

### Improved morphology and crystallinity of fullerene film

SEM was used to examine the surface morphology of the C_60_-SAM film after silane functionalization. As shown in Fig. 2b, some aggregates can be seen on the non-crosslinked C_60_-SAM film, while the silane-crosslinked C_60_-SAM film seems to be smoother, compact and uniform (Fig. 2c). The aggregates found on the C_60_-SAM film may be caused by the intermolecular hydrogen bonding between –COOH groups during baking. When silane was introduced during thermal annealing of C_60_-SAM, most of the hydrogen bonds were preferably formed between –OH groups of the silane molecule and –COOH groups of the C_60_-SAM due to the steric effect^34^,^35^, though the intermolecular interaction among neighbouring and closely packed C_60_-SAM molecules may still have formed upon film formation. We hypothesize that the hydrogen bonding (–OH···–COOH) aligns the C_60_-SAM molecules in order, driven by the silane crosslinking process, instead of randomly forming dimers, and it extends the intermolecular interaction for considerable distances, which results in pseudo-polymer type structures. Thus a more uniform and compact C_60_-SAM film is produced, which was revealed by the SEM study shown in Fig. 2c. To further verify that the ordering of C_60_-SAM can be induced by the crosslinking functionalization, we performed X-ray diffraction measurements of the C_60_-SAM films on glass substrates with and without crosslinking treatment. Figure 2d clearly shows three diffraction peaks including a sharp diffraction peak at 12.4° in the X-ray diffraction pattern of the crosslinked C_60_-SAM film, whereas there is no obvious diffraction peak in the non-crosslinked C_60_-SAM film. These three peaks can be indexed as the (111), (220) and (311) diffraction peaks from a face-centered cubic (fcc) lattice^36^,^37^,^38^, indicating that C_60_-SAM have the same fcc structure as C_60_. It is worth noting that among all possible crystal structures of C_60_, the fcc structure has the best electron mobility^39^. The better crystallization of crosslinked C_60_-SAM supports our hypothesis of good alignment of C_60_-SAM by crosslinking treatment, and explains the improved device open circuit voltage (*V*_OC_) as shown below^17^.

To evaluate the water-resistivity change of the C_60_-SAM-covered perovskite films by crosslinking, we first tested the contact angles of the C_60_-SAM layers deposited on perovskite films with and without crosslinking. The contact angle of the C_60_-SAM covered perovskite film without crosslinking was as low as 46°. The colour of the water droplet changed to yellow after 1–2 s as shown in Fig. 2e, indicating a quick decomposition of MAPbI_3_ to PbI_2_. In contrast, the water resistance of the crosslinked C_60_-SAM film with hydrophobic –CF_3_ functional groups was significantly improved, showing a much larger contact angle of 86° (Fig. 2f) and delayed film colour change up to 120 seconds upon contacting water. These results confirmed the successful functionalization of the C_60_-SAM film by the introduction of the silane agent, which significantly enhanced water-resistivity.

### Doping and crosslinking effect

**Figure 3:**
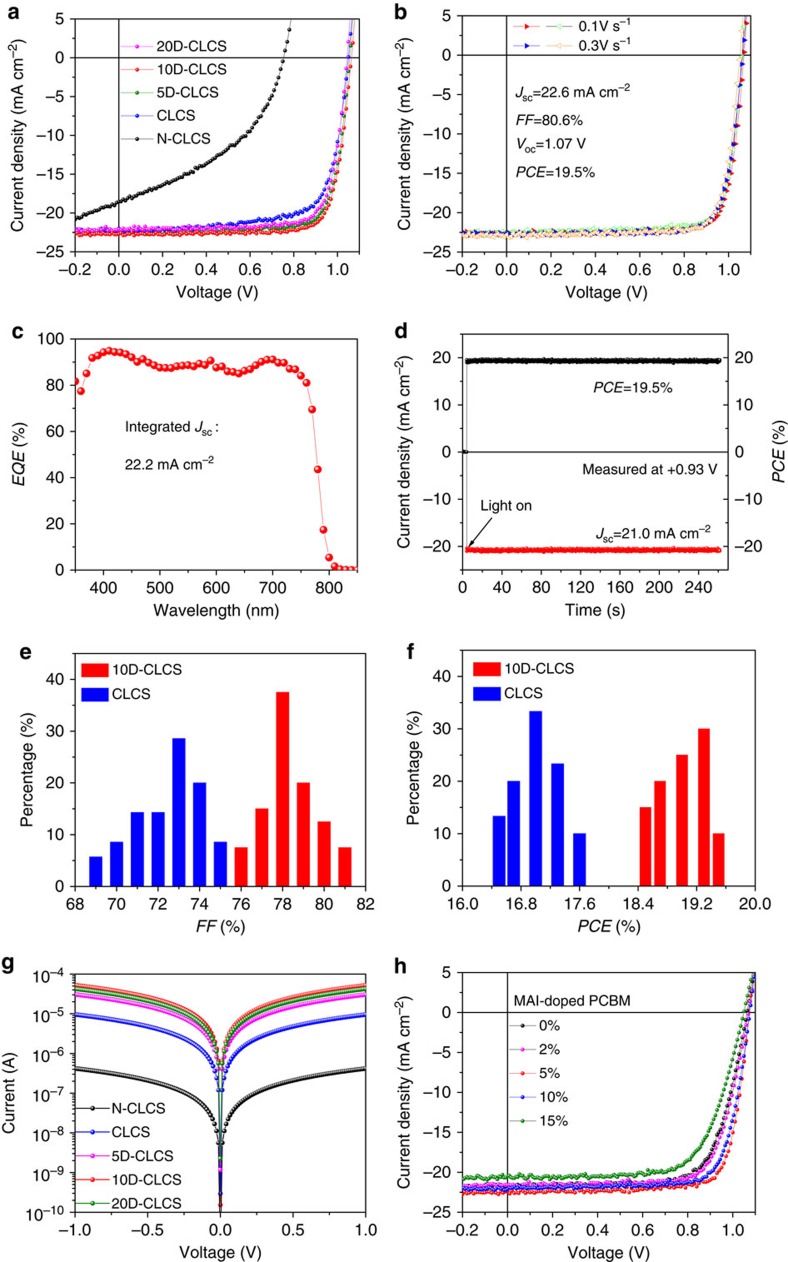
Performance characterizations of the perovskite devices. (**a**) *J*–*V* curves of perovskite solar cells based on different fullerene ETLs. CLCS, crosslinked C_60_-SAM; N-CLCS, non-crosslinked C_60_-SAM; 5D-CLCS, 5 wt.% MAI-doped crosslinked C_60_-SAM; 10D-CLCS, 10 wt.% MAI-doped crosslinked C_60_-SAM; 20D-CLCS, 20 wt.% MAI-doped crosslinked C_60_-SAM. (**b**) *J*–*V* curves with different sweeping directions and sweeping rates (hollow triangles and solid triangles represent the scanning direction from negative to positive bias and from positive to negative bias, respectively). (**c**) *EQE* (the integrated current density is 22.2 mA cm^−2^) and (**d**) steady-state photocurrent and efficiency at the maximum power point (0.93 V) of the optimized perovskite device with 10D-CLCS ETL. Statistics of the fill factor (*FF)* (**e**) and power conversion efficiency (*PCE*). (**f**) Distribution for devices with CLCS (35 samples) and 10D-CLCS ETLs (40 samples). (**g**) Comparison of the *I*–*V* characteristics of N-CLCS, CLCS and 5D-CLCS, 10D-CLCS and 20D-CLCS films deposited on normal glass substrates obtained by four-probe conductivity measurement. (**h**) *J*–*V* curves of perovskite device fabricated with PCBM and MAI-doped PCBM with different doping levels.

We characterized the performance of the devices before and after silane-functionalization of the C_60_-SAM layer. It is noted that a relatively thick fullerene layer is needed to enhance the water resistivity, while the larger thickness and cross-linking process inevitably increases the device contact resistance at the cathode side. To improve the electron extraction capability of the crosslinked C_60_-SAM ETL, a small amount of MAI was blended in the C_60_-SAM as n-dopant via the electron transfer between the anions of the n-dopants and C_60_-SAM^40^,^41^. Lewis base anions (I^−^ from MAI dopants) act as electron donors. Initially the electron density is localized on iodide when two counter ions are effectively separated from each other. As the iodide approaches fullerene upon solvent drying, the energy decreases and the electron density starts to redistribute over iodide to fullerene, which enhances the free electron density of the C_60_-SAM matrix to achieve higher conductivity^40^,^42^. Figure 3a shows the impact of crosslinking and doping of C_60_-SAM layer on the photocurrent density–voltage (*J*–*V*) curves of the corresponding perovskite solar cells. A typical perovskite device with a non-crosslinked C_60_-SAM layer had a short-circuit current density (*J*_SC_) of 18.6 mA cm^−2^, a *V*_OC_ of 0.75 V and a fill factor (*FF*) of 43% yielding a very low *PCE* of 6.0%, which may be explained by the bad morphology as well as the bad electron transport property of the C_60_-SAM layer. When the C_60_-SAM layer was crosslinked, the device *V*_OC_ and *FF* increased dramatically to 1.05 V and 73.9%, respectively. The device using crosslinked C_60_-SAM delivered an efficiency of 17.0%, which is approaching that of the devices made with conventional PCBM^17^,^27^,^43^. MAI as n-dopant was then introduced into the crosslinked C_60_-SAM layer, and the changed conductivity was found to have a significant impact on the device performance, particularly *FF*. After doping the crosslinked C_60_-SAM with 10 wt.% MAI, the device showed a decent *FF* of 80.0%, resulting in a significantly enhanced *PCE* of 19.3%. Further increasing doping ratio to 20 wt.% deteriorated the device performance. All the photovoltaic parameters of the perovskite devices based on non-crosslinked C_60_-SAM, crosslinked C_60_-SAM and crosslinked C_60_-SAM with different MAI doping levels are summarized in Table 1. As shown in Fig. 3b, the optimized device based on doped crosslinked C_60_-SAM exhibited a *J*_SC_ of 22.6 mA cm^−2^, a *V*_OC_ of 1.07 V and a *FF* of 80.6%, yielding a *PCE* of 19.5%. No obvious photocurrent hysteresis was observed in the device made with doped and crosslinked C_60_-SAM by changing the sweeping direction and sweeping rate (Fig. 3b), indicating the effective fullerene passivation^26^. The integrated *J*_SC_ from external quantum efficiency (*EQE*) spectrum shown in Fig. 3c reached 22.2 mA cm^−2^, which is in good agreement with that from *J*–*V* measurement. The steady-state photocurrent and efficiency measured at the maximum power point (0.93 V) are presented in Fig. 3d, which confirms the device performance parameters extracted from the *J*–*V* curve and verifies the absence of photocurrent hysteresis in our devices. Prolonged steady-state performance up to 2,000 s can be found in Supplementary Fig. 1. The statistics of *FF* and *PCE* distributions shown in Fig. 3e,f demonstrate the reliability and repeatability of the *FF* and *PCE* enhancement obtained by the crosslinking and doping effect.

## Discussion

The significant enhancement of *FF* to over 80% can be explained by the reduced contact (or series) resistance (*R*_S_) by doping the C_60_-SAM layer. The series resistance of devices with different ETLs was derived by fitting the slope of *J*–*V* curves. As shown in Table 1, the device *R*_S_ was reduced from 8.19 Ω cm^2^ to 3.72 Ω cm^2^ simply by crosslinking the C_60_-SAM. *R*_S_ was further reduced to 2.08 Ω cm^2^ via doping 10 wt.% of MAI into crosslinked C_60_-SAM. To verify the increased conductivity through crosslinking and doping, conductivity of the C_60_-SAM films was measured with co-planar films made on glass. The obtained current–voltage curves were presented in Fig. 3g. The non-crosslinked C_60_-SAM film showed a quite low conductivity of 8.4 × 10^-5^ S cm^−1^. After silane treatment, the conductivity of crosslinked C_60_-SAM increased significantly to 1.9 × 10^−3^ S cm^−1^. By introducing 10 wt.% of MAI for n-doping, the conductivity was further boosted to 1.1 × 10^−2^ S cm^−1^, which is over 100 times higher than that of non-crosslinked C_60_-SAM. Thus, the conductivity variation follows the same trend with that derived from *J*–*V* curves, though a different increasing factor was observed here, which can be explained by the different transport processes measured. The conductivity enhancement by crosslinking C_60_-SAM can be explained by the significantly improved film morphology. A more compact packing/connection of C_60_-SAM by crosslinking is expected to reduce activation energy (*E*_a_) for the hopping of electrons between the C_60_-SAM molecules^44^,^45^,^46^. In addition, the extended intermolecular overlap through hydrogen bonding in the crosslinked C_60_-SAM layer increased the electron density of the aromatic system of the C_60_-SAM molecules^44^, which produced a substantial decrease in *E*_a_. It should be noted that the doping of C_60_-SAM does not necessarily need to be coupled with the crosslinking to enhance the device performance. In devices with PCBM rather than C_60_-SAM as ETL, we also observed an enhanced efficiency to 19.1% by simply doping PCBM with 5 wt.% of MAI, as shown in Fig. 3h. Detailed photovoltaic parameters can be found in Supplementary Table 1. This doping also explained the recently observed very large *FF* devices where excess amount of MAI was used in fabricating the perovskite films which simultaneously doped PCBM^47^,^48^.

**Figure 4:**
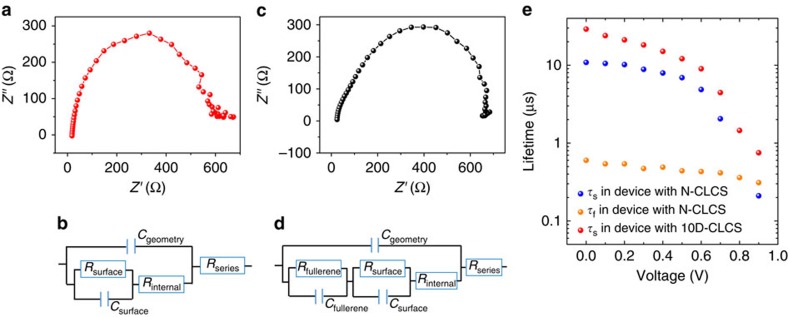
Recombination lifetime analysis. Typical impedance spectra of perovskite devices with (**a**) 10D-CLCS and (**c**) N-CLCS. The corresponding equivalent circuits of perovskite devices with (**b**) 10D-CLCS and (**d**) N-CLCS for impedance spectroscopy fitting. (**e**) Recombination lifetime of different recombination processes extracted from the impedance spectroscopy modelling. The recombination lifetime data in red are extracted from the device with 10D-CLCS, while the recombination lifetime data in blue and orange are extracted from the device with N-CLCS.

To understand how doping and crosslinking of C_60_-SAM boosts the perovskite photovoltaic performance, the recombination dynamics process was examined by impedance spectroscopy (IS) measurement, which was widely utilized in analysing many photovoltaic cells, such as dye-sensitized solar cells^49^,^50^, organic solar cells^51^ and perovskite solar cells^26^,^52^,^53^. The measured impedance spectra of devices with non-crosslinked C_60_-SAM and doped crosslinked C_60_-SAM present different characteristics. As shown in Fig. 4a, the impedance spectrum of the devices with doped crosslinked C_60_-SAM have one semicircle, indicating only one recombination channel dominates in these devices. It could be assigned to the traps-assisted charge recombination at the film surface or grain boundaries because the perovskite thin films have excellent crystallinity and low density of traps^54^. The equivalent circuit used for these devices is shown in Fig. 4b. In sharp contrast, the impedance spectrum of the devices with non-crosslinked C_60_-SAM show two semicircles with different time constants (Fig. 4c), indicating that two recombination channels exist in these devices. Besides the surface or grain boundary recombination, the large density of traps in C_60_-SAM is most likely the origin of the additional recombination process. As a result, an additional resistor–capacitor (RC) circuit was added into the equivalent circuit (Fig. 4d) to separate the individual contribution of the two recombination channels. Figure 4e shows the recombination lifetime of different recombination channels extracted from the IS modelling. The fitted values from impedance spectra of perovskite devices with 10D-CLCS and N-CLCS can be found in Supplementary Fig. 2. For the devices with non-crosslinked C_60_-SAM, the shorter time constant (*τ*_f_) was assigned to the recombination process in the C_60_-SAM, because it is almost invariant to the applied bias and mainly determined by the defects in C_60_-SAM. The longer time constant (*τ*_s_) was assigned to the recombination process in perovskite. Apparently, the device with doped and crosslinked C_60_-SAM has much longer recombination lifetime, proving a better passivation effect by crosslinking C_60_-SAM.

**Figure 5:**
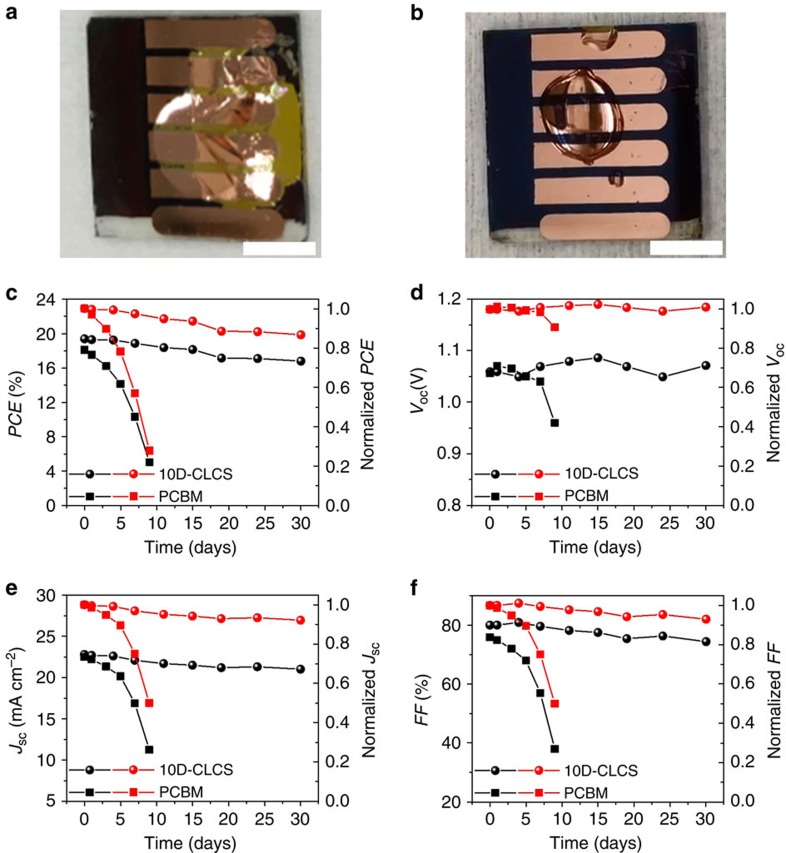
Water-resistivity and shelf-stability of the perovskite devices without encapsulation in ambient environment. Images of the devices based on conventional PCBM (**a**) and 10D-CLCS ETLs (**b**) after exposure to water droplet for 4 min. The scale bars in both the images are 0.5 cm. Photovoltaic performance (black curves) and normalized performance (red curves) of the typical perovskite devices with PCBM and 10D-CLCS ETLs in an ambient environment without encapsulation as a function of storage time. (**c**) *PCE*; (**d**) open-circuit voltage (*V*_OC_); (**e**) short-circuit current density (*J*_SC_); and (**f**) *FF*.

The main motivation to crosslink the C_60_-SAM layer is to enhance the water resistance of the perovskite devices, though we did see an enhancement of device performance simultaneously. We first tested the stability of these devices under extreme conditions by dropping water on top of the devices for a certain time. Figure 5a,b shows the photograph (extracted from Supplementary Movie 1) of devices based on conventional PCBM and crosslinked C_60_-SAM ETLs after exposure to water droplets for 4 min. It is noted that the perovskite device with conventional PCBM completely decomposed in 30 s after dropping water on it, while the device with crosslinked C_60_-SAM was much more stable and no colour change was observed over 4 min, indicating no decomposition of perovskite to PbI_2_ occurred. More details can be found in the Supplementary Movie 1. Thereafter, we monitored the stability of perovskite devices using both conventional PCBM and self-developed doped crosslinked C_60_-SAM as a function of storage time in air. All the devices were stored without encapsulation in an ambient environment. The original *J*–*V* curves showing the degradation can be found in Supplementary Fig. 3 for both types of perovskite devices stored at ambient environment for various days. As shown in Fig. 5c, in terms of the *PCE* of typical devices based on the conventional PCBM, the majority of initial performance was lost dramatically in 1 week. While the typical perovskite devices using doped crosslinked C_60_-SAM demonstrated encouragingly much better stability and maintained almost 90% of the original device *PCE* after 30 days. The variation of key photovoltaic parameters versus storage time is summarized in Fig. 5d–f. We also carried out a stability study of the unsealed perovskite devices with both PCBM and crosslinked C_60_-SAM ETL under continuous illumination (20 mW cm^−2^) in highly humid air (50–75% relative humidity). The devices were removed at an interval of 24 h to record the photovoltaic performance. The variation of key photovoltaic parameters versus illumination time is summarized in Supplementary Fig. 4. The original *J*–*V* curves showing the degradation can be found in Supplementary Fig. 5 for both types of perovskite devices under continuous light illumination in humid air. As shown in the Supplementary Fig. 4, a typical device based on the conventional PCBM lost the majority of initial performance in 36 h. In striking contrast, the typical perovskite devices using crosslinked and doped C_60_-SAM demonstrated encouragingly much better stability and still maintained roughly 70% of the original device *PCE* even after 168 h under light soaking. This represents a significant improvement of device performance compared with what was reported. Previous study on the stability of unsealed perovskite devices under continuous light illumination in humid air showed that the photovoltaic performance usually degraded quickly to less than 70% of their original efficiency within 48 h (refs 25, 55, 56). Such exceptional ambient stability can be attributed to the crosslinkable silane functionalized and doped fullerene, which forms an internal stable hydrophobic encapsulating layer while maintaining excellent electron transporting properties as a result of n-doping.

In summary, we reported a specific crosslinked C_60_-SAM ETL by bonding crosslinkable silane coupling agent, which have hydrophobic functional groups, onto C_60_-SAM. Such ETL successfully blocks any accessible pathways of water molecule permeation and thus protect the perovskite films underneath from moisture-caused damage. The results show that the crosslinking process can also improve the transport properties of ETL. In addition, MAI was demonstrated as an effective n-dopant for C_60_-SAM to further enhance its conductivity. The perovskite devices fabricated with doped and crosslinked C_60_-SAM exhibit much longer recombination lifetime in comparison to non-crosslinked C_60_-SAM, yielding a high efficiency of 19.5% without photocurrent-hysteresis. More encouragingly, these devices show significantly improved stability and the majority (nearly 90%) of their original high efficiency can be retained after exposing to an ambient environment for 30 days. This work paves the way towards addressing the main hindrance to the practical application of perovskite devices under ambient conditions.

## Methods

### Film formation and device fabrication

Methylammonium iodide (CH_3_NH_3_I, MAI) was synthesized using the method described in our previous publication^30^. Poly(bis(4-phenyl)(2,4,6-trimethylphenyl)amine) (PTAA) film as HTL was first deposited on cleaned ITO substrates by spin coating 0.25 wt.% PTAA solution at 6,000 r.p.m., and the as-prepared film was thermally annealed at 100 °C for 10 min (ref. 27). The MAPbI_3_ films were fabricated by solvent annealing-induced interdiffusion method^30^. PbI_2_ (dissolved in *N*,*N*-dimethylformamide) was spin-coated on top of PTAA/ITO substrate at 6,000 r.p.m. for 35 s. Then MAI (dissolved in 2-propanol) was spin-coated on top of the dried PbI_2_ layer at room temperature at 6,000 r.p.m. for 35 s. Afterwards the stacked precursor layers were solvent-annealed at 100 °C for 1 h (ref. 10). For the control devices using conventional PCBM as ETLs, the PCBM (dissolved in 1,2-Dichlorobenzene (DCB), 2 wt.%) was spin-coated on top of the perovskite layer at 6,000 r.p.m. for 35 s and annealed at 100 °C for 60 min. To develop crosslinked fullerene as stable and efficient ETLs, C_60_-substituted benzoic acid self-assembled monolayer (C_60_-SAM) material (1-Materials) was selected as the starting material. C_60_-SAM (2 wt.%) was dissolved in a 1:1 volume ratio of tetrahydrofuran: DCB mixture solvent and the solution was then passed through a 0.2 μm PTFE filter. The C_60_-SAM solution was spin-coated on top of the perovskite films at 6,000 r.p.m. for 35 s. Subsequently, all the films were put on a hotplate and covered by a glass petri dish. For the devices with crosslinked C_60_-SAM, 5-8 μl of trichloro(3,3,3-trifluoropropyl)silane (Sigma-Aldrich) was added at the edge of the petri dish during the thermal annealing process (100 °C for 60 min). The silane vapour was expected to be able to penetrate into the C_60_-SAM layer and crosslink with C_60_-SAM. C_60_ (20 nm) and bathocuproine (8 nm) were then thermally evaporated as the buffer layers. The devices were completed by thermal evaporation of Cu (80 nm) as the top electrode. The device working area was 8 mm^2^, as defined by the overlap of the ITO substrate and the Cu cathode. A small amount (5–20 wt.%) of MAI (pre-dissolved in 2-propanol) was blended in the C_60_-SAM solution before spin-coating to dope it.

### Film and device characterization

The photocurrent density–voltage (*J*–*V*) curves of the devices were measured under AM 1.5 G irradiation (100 mW cm^−2^), which was produced by a xenon-lamp based solar simulator (Oriel 67005, 150 W Solar Simulator). The light intensity was calibrated by a Schott visible-colour glass-filtered (KG5 colour-filtered) Si diode (Hamamatsu S1133) before photocurrent measurement. Keithley 2400 Source-Meter was used for recording the *J*–*V* measurements. The *J*–*V* testing was performed with forward scan direction (if not specified in the manuscript) at 0.1 V s^−1^ and sweep delay time was 50 ms. No preconditioning protocol was used before the characterization. A shadow mask with an aperture area of 6.44 mm^2^ was used for the photocurrent calibration (Supplementary Fig. 6 and Supplementary Table 2). The steady-state *PCE* was measured by recording the photocurrent at a bias voltage of 0.93 V. We calibrated the measured photocurrent density with integrated *J*_sc_ from *EQE* measurement. Calibration with Oriel Si detector was carefully carried out before *EQE* measurement. The *EQE* was obtained using a Newport QE measurement kit by focusing a monochromatic beam of light onto the devices. The X-ray diffraction patterns of C_60_-SAM films were obtained by a Rigaku D/Max-B X-ray diffractometer in the Bragg–Brentano parafocusing geometry. A conventional cobalt target X-ray tube equipped in the diffracted-beam monochromator was set to 40 kV and 30 mA. Impedance spectroscopy of the device was measured by the E4980A Precision LCR Meter from Agilent with homemade software under 1 sun illumination at room temperature. The resistivity measurements of various C_60_-SAM films were performed using a four-point probe setup as described in the previous work^43^. C_60_-SAM films were deposited on normal glass substrates following the same procedure in device fabrication described above and Cu electrodes with 80 nm thickness were then thermal evaporated onto the resultant C_60_-SAM films through a shadow mask. C_60_-SAM films before and after crosslinking were scratched off the substrates for FTIR (Fourier transform infrared spectroscopy) analysis. The FTIR spectra of C_60_-SAM powder were collected in the transmittance mode on the PerkinElmer IR spectrometer instrument in the 400–4,000 cm^−1^ region. The SEM images were taken from a Quanta 200 FEG environmental scanning electron microscope. High resolution transmission electron microscopy analysis was carried out on a FEI OSIRIS microscope. The cross-sectional samples were prepared by using focused-ion-beam equipment (FEI Helios 660).

### Data availability

The data that support the findings of this study are available from the corresponding author upon request.

## Additional information

**How to cite this article:** Bai, Y. *et al*. Enhancing stability and efficiency of perovskite solar cells with crosslinkable silane-functionalized and doped fullerene. *Nat. Commun.* 7:12806 doi: 10.1038/ncomms12806 (2016).

## Supplementary Material

Supplementary Information Supplementary Figures 1 - 6 and Supplementary Tables 1 and 2

Supplementary Movie 1 Devices based on (left) conventional PCBM and (right) crosslinked C60-SAM ETLs were exposed to water droplets for over 4 minutes. Device with conventional PCBM completely decomposed in 30 seconds after dropping water on it, while no color change was observed over 4 minutes for the device with crosslinked C60-SAM.

## Figures and Tables

**Table 1:** Summary of the best photovoltaic performance of the perovskite solar cells using different ETLs.

**ETLs**	***J***_**SC**_**(mA** **cm**^**−2**^**)**	***V***_**OC**_**(V)**	*FF*(%)	*PCE*(%)	***R***_**S**_ **(Ω** **cm**^**2**^**)**
N-CLCS	18.6	0.75	43.0	6.0	8.19
CLCS	21.9	1.05	73.9	17.0	3.72
5D-CLCS	22.5	1.06	78.5	18.7	3.12
10D-CLCS	22.7	1.06	80.0	19.3	2.08
20D-CLCS	22.1	1.04	78.9	18.1	2.91

*R*_S_ is the device series resistance derived from *J*–*V* curves.
