# Supplementary material for: Enhancing stability and efficiency of perovskite solar cells with crosslinkable silane-functionalized and doped fullerene
Source: Nat Commun. 2016 Oct 5;7:12806. doi: 10.1038/ncomms12806 (PMC5059465; doi:10.1038/ncomms12806)
Supplement: Supplementary Information — Supplementary Figures 1 - 6 and Supplementary Tables 1 and 2 [file ncomms12806-s1.pdf]

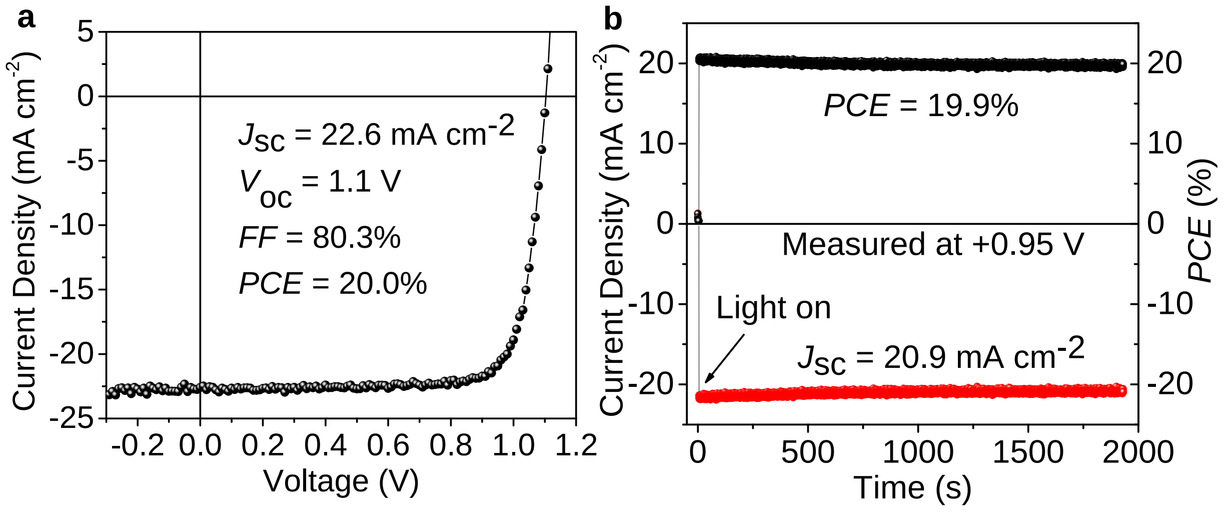

Supplementary Figure 1 Performance of optimized perovskite device with 10D-CLCS: (a) *J-V* curves and (b) steady-state photocurrent and efficiency at the maximum power point (0.95 V) of the optimized perovskite device with 10D-CLCS ETL.

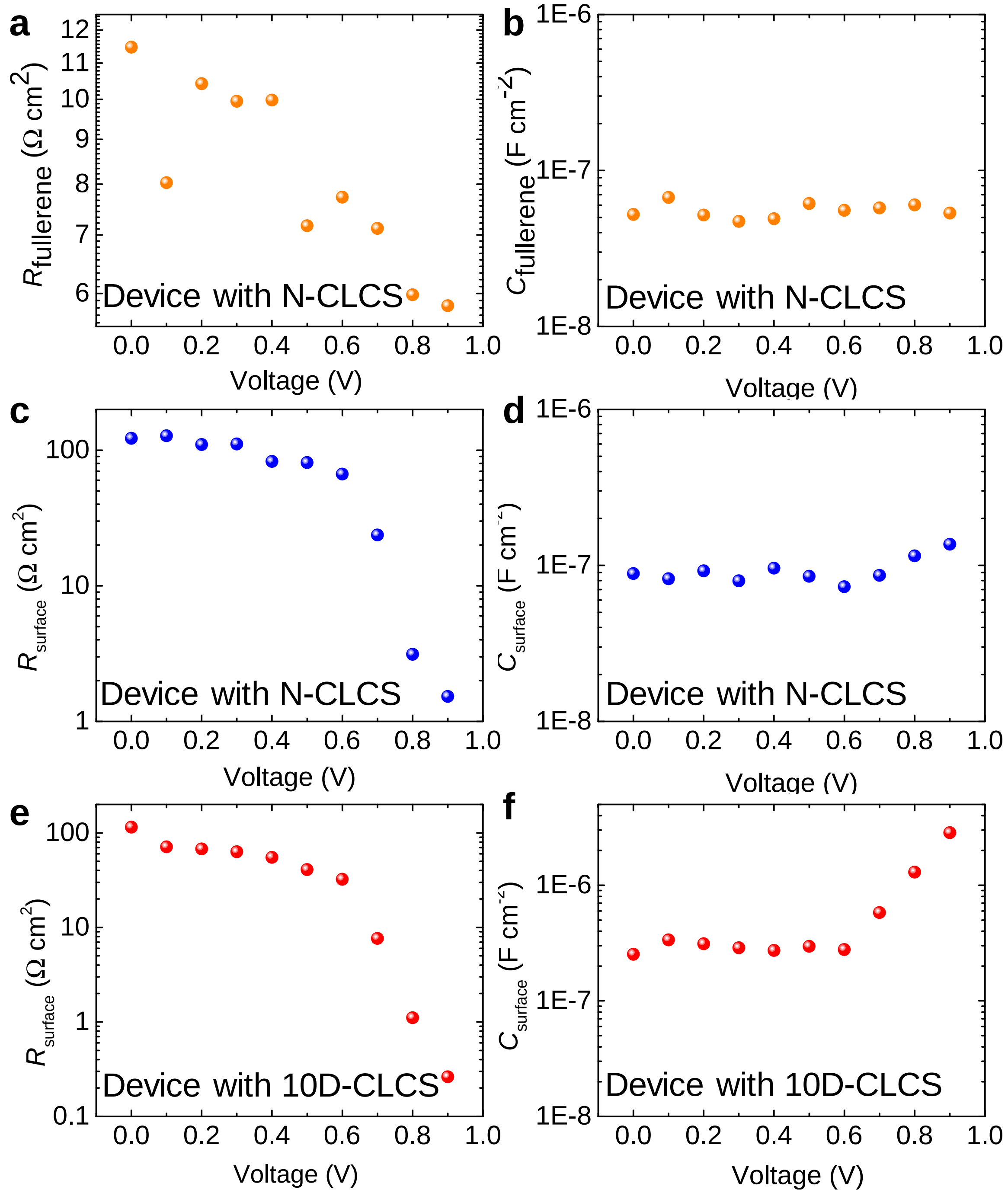

Supplementary Figure 2: The fitted values from impedance spectra of perovskite devices with 10D-CLCS and N-CLCS: (a) $R_{\text{fullerene}}$ for the device with N-CLCS; (b) $C_{\text{fullerene}}$ for the device with N-CLCS; (d) R_{surface} for the device with N-CLCS; (e) C_{surface} for the device with N-CLCS; (e) R_{surface} for the device with 10D-CLCS; (f) C_{surface} for the device with 10D-CLCS.

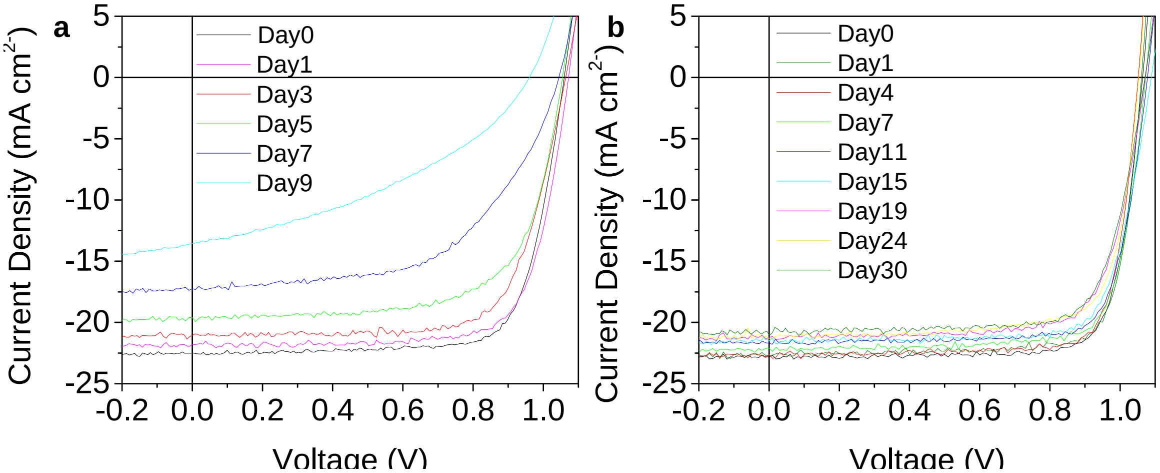

Supplementary Figure 3 Comparison of device degradation stored in dark: *J*-*V* curves of typical perovskite device fabricated with PCBM (a) and 10D-CLCS (b) ETLs stored in ambient air for various days.

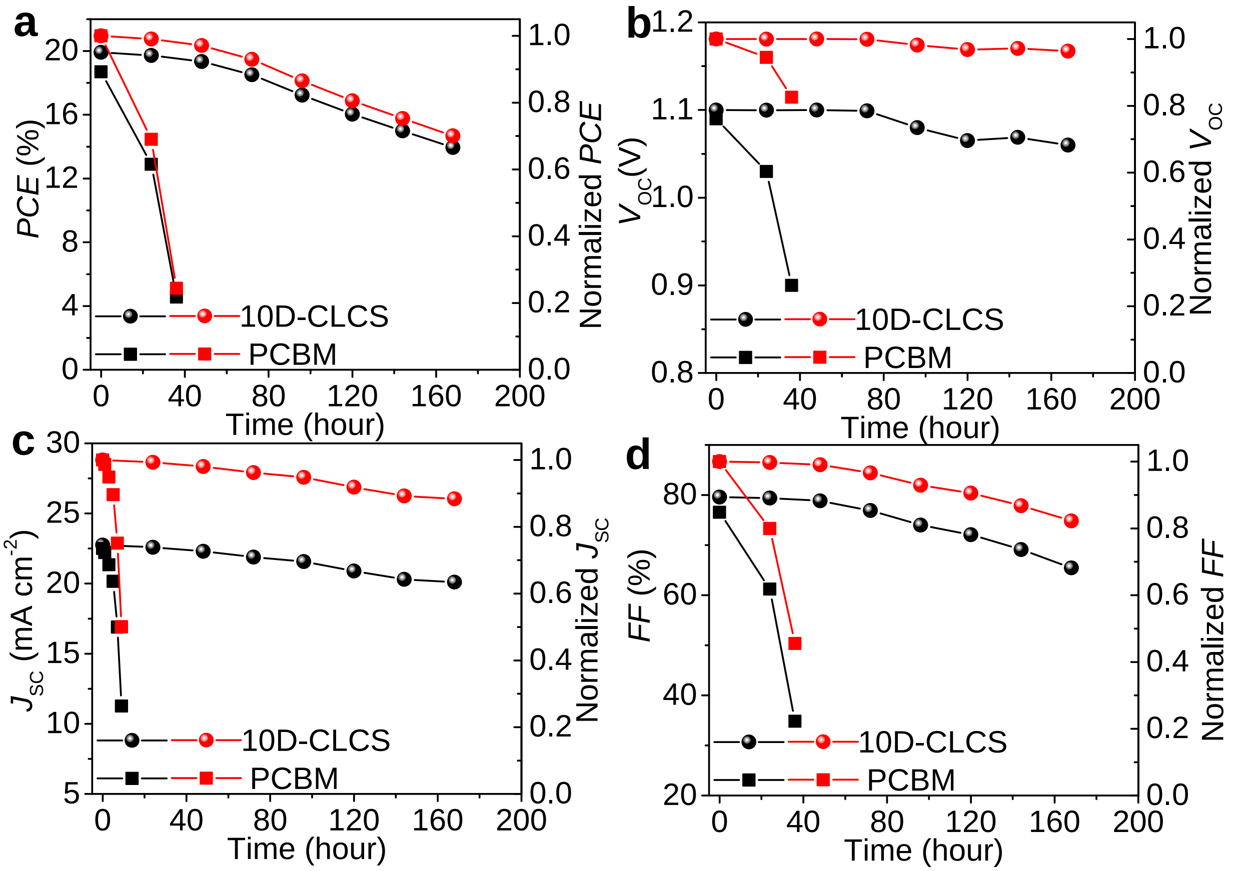

Supplementary Figure 4 Device stability under continuous light illumination: Photovoltaic performance (black curves) and normalized performance (red curves) of the typical unsealed perovskite devices with PCBM and 10D-CLCS ETLs under continuous 20 mW cm^{2-} simulated solar light illumination in humid air as a function of illumination time. (a) *PCE*; (b) V_{OC}; (c) J_{SC}; and (d) *FF*.

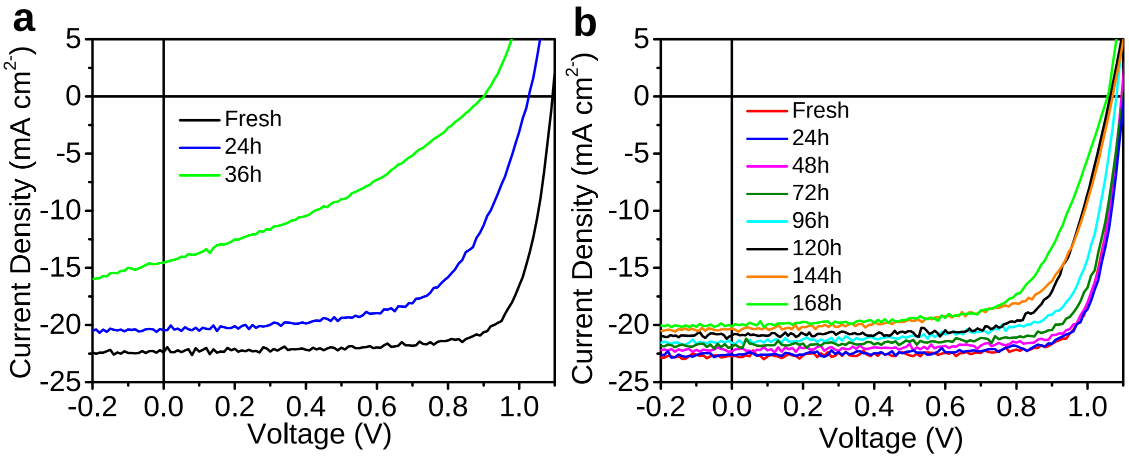

Supplementary Figure 5 Comparison of device degradation under light illumination: *J-V* curves of typical perovskite device fabricated with PCBM (a) and 10D-CLCS (b) ETLs under continuous light illumination in humid air for various time.

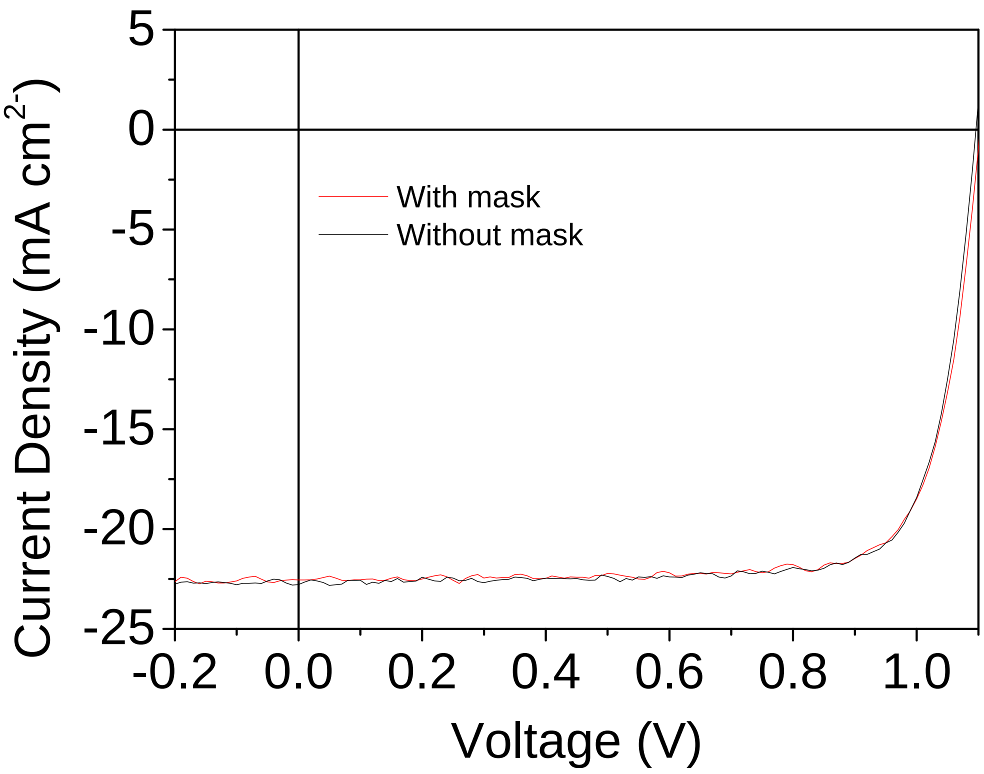

Supplementary Figure 6 Photocurrent calibration with mask: *J-V* curves of typical perovskite device fabricated with 10D-CLCS ETL measured with and without mask.

Supplementary Table 1 Performance of perovskite devices based undoped and doped PCBM: Photovoltaic parameters of perovskite solar cells employing PCBM and MAI-doped PCBM with different doping ratios.

Doping ratio (wt%)	J_{SC} (mA cm^{-2})	V_{OC} (V)	*FF* (%)	*PCE* (%)
0	21.7	1.06	72.1	16.6
2	21.6	1.07	74.0	17.1
5	22.4	1.07	77.6	19.1
10	22.1	1.07	76.7	18.1
15	20.5	1.05	69.3	14.9

Supplementary Table 2 Performance calibration with mask: Photovoltaic parameters of perovskite solar cells fabricated with 10D-CLCS ETL measured with and without mask.

	J_{SC} (mA cm^{-2})	V_{OC} (V)	*FF* (%)	*PCE* (%)
Without Mask	22.8	1.1	78.9	19.8
With Mask	22.5	1.1	79.4	19.7
